# Colorectal Cancer-Associated Immune Exhaustion Involves T and B Lymphocytes and Conventional NK Cells and Correlates With a Shorter Overall Survival

**DOI:** 10.3389/fimmu.2021.778329

**Published:** 2021-12-16

**Authors:** Carlo Sorrentino, Luigi D’Antonio, Cristiano Fieni, Stefania Livia Ciummo, Emma Di Carlo

**Affiliations:** ^1^ Department of Medicine and Sciences of Aging, “G. d’Annunzio” University” of Chieti-Pescara, Chieti, Italy; ^2^ Anatomic Pathology and Immuno-Oncology Unit, Center for Advanced Studies and Technology (CAST), “G. d’Annunzio” University of Chieti-Pescara, Chieti, Italy

**Keywords:** colorectal cancer, immune exhaustion genes, immune checkpoints, T cell exhaustion, B cell exhaustion, innate lymphoid cells, conventional NK cells

## Abstract

Colorectal cancer (CRC) is one of the most common cancer worldwide, with a growing impact on public health and clinical management. Immunotherapy has shown promise in the treatment of advanced cancers, but needs to be improved for CRC, since only a limited fraction of patients is eligible for treatment, and most of them develop resistance due to progressive immune exhaustion. Here, we identify the transcriptional, molecular, and cellular traits of the immune exhaustion associated with CRC and determine their relationships with the patient’s clinic-pathological profile. Bioinformatic analyses of RNA-sequencing data of 594 CRCs from TCGA PanCancer collection, revealed that, in the wide range of immune exhaustion genes, those coding for *PD-L1*, *LAG3* and *T-bet* were associated (Cramér’s V=0.3) with MSI/dMMR tumors and with a shorter overall survival (log-rank test: *p=*0.0004, *p*=0.0014 and *p*=0.0043, respectively), whereas high levels of expression of *EOMES*, *TRAF1*, *PD-L1*, *FCRL4, BTLA* and *SIGLEC6* were associated with a shorter overall survival (log-rank test: *p*=0.0003, *p*=0.0188, *p*=0.0004, *p*=0.0303, *p*=0.0052 and *p*=0.0033, respectively), independently from the molecular subtype of CRC. Expression levels of *PD-L1, PD-1, LAG3, EOMES, T-bet*, and *TIGIT* were significantly correlated with each other and associated with genes coding for *CD4^+^* and *CD8^+^CD3^+^* T cell markers and *NKp46^+^CD94^+^EOMES^+^T-bet^+^* cell markers, (OR >1.5, *p*<0.05), which identify a subset of group 1 innate lymphoid cells, namely conventional (c)NK cells. Expression of *TRAF1* and *BTLA* co-occurred with both T cell markers, *CD3γ, CD3δ, CD3ε, CD4*, and B cell markers, *CD19, CD20* and *CD79a* (OR >2, *p*<0.05). Expression of *TGFβ1* was associated only with *CD4* ^+^ and *CD8^+^CD3ε^+^* T cell markers (odds ratio >2, *p*<0.05). Expression of *PD-L2* and *IDO1* was associated (OR >1.5, *p*<0.05) only with cNK cell markers, whereas expression of *FCRL4, SIGLEC2* and *SIGLEC6* was associated (OR >2.5; *p*<0.05) with *CD19^+^CD20^+^CD79a^+^* B cell markers. Morphometric examination of immunostained CRC tissue sections, obtained from a validation cohort of 53 CRC patients, substantiated the biostatistical findings, showing that the highest percentage of immune exhaustion gene expressing cells were found in tumors from short-term survivors and that functional exhaustion is not confined to T lymphocytes, but also involves B cells, and cNK cells. This concept was strengthened by CYBERSORTx analysis, which revealed the expression of additional immune exhaustion genes, in particular *FOXP1, SIRT1, BATF, NR4A1* and *TOX*, by subpopulations of T, B and NK cells. This study provides novel insight into the immune exhaustion landscape of CRC and emphasizes the need for a customized multi-targeted therapeutic approach to overcome resistance to current immunotherapy.

## Introduction

Colorectal cancer (CRC) ranks third for incidence, after breast and lung cancers, and second for mortality, after lung cancer (1). Mortality is mainly due to metastatic disease, which affects nearly 22% of CRC patients at diagnosis, and more than 50% thereafter due to relapses (2). CRC is an age-related malignancy, since 90% of cases are diagnosed after the age of 50, and the average age at diagnosis is 70 (1, 3), therefore the overall aging of the population, will inevitably lead to an increase in the number of new cases diagnosed in the near future, which makes CRC a significant public health challenge.

Advances in patient-tailored therapy, which is based on the mutational, transcriptomic, and proteomic profiles of individual CRC (4), and on its immune cell context, can fulfill this need. Genetic and epigenetic alterations play a key role in shaping the CRC microenvironment (5). Defects in DNA mismatch repair proteins and subsequent microsatellite instability lead to the accumulation of mutation loads in cancer-related genes and the generation of neoantigens, which promote intra-tumoral immune cell influx, essential for immunotherapy. Mismatch repair deficiency (dMMR) or microsatellite instability (MSI) is significantly associated with long-term immunotherapy-related responses and better prognosis in cancers treated with immune checkpoint inhibitors (6). However, many of the patients that initially respond to treatment later become resistant or relapse due to the occurrence of further genetic and epigenetic alterations, which generate new cancer clones with different molecular traits. These molecular events re-shape the tumor microenvironment (TME), upregulating other inhibitory receptors and immunosuppressive mediators, and expanding the landscape of immune cell exhaustion. Exhaustion is a functional status attributed to T lymphocytes and characterized by the loss of effector function, including proliferation, release of cytokines, and secretion of cytolytic molecules, due to persistent antigen stimulation and inflammatory signals in pathological contexts, such as chronic infections or inflammatory diseases and cancer (7, 8). However, in the last few years it has become progressively evident that exhaustion is not exclusive of T cells, but can also affect other immune cell subsets (9–12) homing the TME.

In this study, using bioinformatics, we analyzed the transcriptional profile, and concomitant immune cell context, of the immune exhaustion in CRCs of the TCGA PanCancer collection, and its relationship with patients’ clinical-pathological characteristics and follow-ups. A validation cohort of CRC patients, which includes short- and long-term survivors, provided tumor samples to localize and quantify, by immunohistochemistry and computerized morphometric analysis, immune exhaustion gene expression at protein level.

Our findings reveal that high levels of expression of specific immune exhaustion genes occurs in MSI molecular subtype, however, independently from the molecular subtype of CRC, high expression levels of a range of immune exhaustion genes regulating different immune cell subsets, are significantly associated with worst prognosis. Biostatistical co-occurrence and morphological co-localization of the expression of immune exhaustion genes and markers of T and B lymphocytes, and of conventional (c) NK cells, strongly suggest that, regardless of the molecular subtype of tumor, CRC-associated immune exhaustion is not only a “T cell matter”. CIBERSORTx analysis corroborates this concept revealing that the expression of relevant immune exhaustion-related genes, detected in the different T lymphocyte subpopulations, also involves B and NK cell subpopulations.

Profiling of functional immune exhaustion in individual CRC can lead to the development of a successful patient tailored therapy to counteract metastasis and disease recurrence.

## Materials and Methods

### Bioinformatic Analyses

For bioinformatic analyses, RNA sequencing (seq) data (obtained using the Illumina HiSeq 2000 RNA Sequencing System, Version 2) of tumor samples from the “*Colorectal Adenocarcinoma TCGA PanCancer collection*”, which includes 594 CRC cases (**Table 1**), were downloaded from the cBioportal for Cancer Genomics database (https://www.cbioportal.org; cBioPortal, RRID : SCR_014555). The expression of the genes listed in the **Tables 2**, **3** was examined.

**Table 1:** Clinicopathological characteristics of patients included in the *Colorectal Adenocarcinoma TCGA PanCancer collection* and in the validation cohort.

	PanCancer collection		Validation cohort	
	Number	Percentage	Number	Percentage
**Sex**				
Female	280	47.30%	24	45.28%
Male	312	52.70%	29	54.72%
*Total*	592*	100.00%	53	100.00%
**Age**				
≤40	19	3.21%	2	3.77%
41-50	63	10.64%	6	11.32%
51-60	106	17.91%	10	18.87%
61-70	164	27.70%	14	26.42%
71-80	163	27.53%	14	26.42%
≥81	77	13.01%	7	13.21%
*Total*	592*	100.00%	53	100.00%
**Clinical stage**				
Stage I	103	17.82%	10	18.87%
Stage II	220	38.06%	20	37.74%
Stage III	170	29.41%	15	28.30%
Stage IV	85	14.71%	8	15.09%
*Total*	578*	100.00%	53	100.00%
**Molecular Subtype^†^**				
CIN^‡^	328	71.46%	38	71.70%
GS^§^	58	12.64%	6	11.32%
MSI^¶^	63	13.73%	7	13.21%
POLE^#^	10	2.18%	2	3.77%
*Total*	459*	100.00%	53	100.00%

*Clinicopathological data were not available for all patients of the PanCancer collection.

^†^Cancer Genome Atlas Network. Comprehensive molecular characterization of human colon and rectal cancer. Nature (2012) 487:330-7.

^‡^Chromosomal instability.

^§^Genomically stable.

^¶^Microsatellite instability.

^#^Polymerase epsilon gene mutation.

**Table 2:** Immune exhaustion-related genes.

Gene Symbol	Protein name
**Inhibitory receptors**	
*BTLA*	B- And T-Lymphocyte Attenuator
*CD22*	CD22 Molecule
*(SIGLEC2)*	(Sialic Acid Binding Ig Like Lectin 2)
*CD274*	CD274 Antigen
*(PD-L1)*	(Programmed Death-Ligand 1)
*CD327*	CD327 Antigen
*(SIGLEC6)*	(Sialic Acid Binding Ig Like Lectin 6)
*CTLA4*	Cytotoxic T-Lymphocyte Associated Protein 4
*FCRL4*	Fc Receptor Like 4
*HAVCR2*	Hepatitis A Virus Cellular Receptor 2
*(TIM-3)*	(T-Cell Immunoglobulin And Mucin-Domain Containing-3)
*LAG3*	Lymphocyte-Activation Gene 3
*(CD223)*	(CD223 Antigen)
*LAIR1*	Leukocyte-Associated Immunoglobulin-Like Receptor 1
*(CD305)*	(CD305 Antigen)
*NR4A1*	Nuclear Receptor Subfamily 4 Group A Member 1
*PDCD1*	Programmed Cell Death 1
*(PD-1)*	
*PDCD1LG2*	Programmed Cell Death 1 Ligand 2
*(PD-L2)*	
*TIGIT*	T Cell Immunoreceptor With Ig And ITIM Domains
*TRAF1*	TNF Receptor Associated Factor 1
*VSIR*	V-Set Immunoregulatory Receptor
*(VISTA)*	(V-Domain Ig Suppressor Of T Cell Activation)
*VTCN1*	V-Set Domain Containing T Cell Activation Inhibitor 1
*(B7H4)*	(B7 Family Member, H4)
**Immunosuppressive mediators**	
*IDO1*	Indoleamine 2,3-Dioxygenase 1
*IFNA1*	IFN-Alpha
*IFNB1*	IFN-Beta
*IL6*	Interleukin 6
*IL10*	Interleukin 10
*TGFB1*	Transforming Growth Factor Beta 1
**Transcription factors**	
*BATF*	Basic Leucine Zipper ATF-Like Transcription Factor
*EOMES*	Eomesodermin
*FOXO1*	Forkhead Box O1
*FOXP1*	Forkhead Box P1
*NFATC1*	Nuclear Factor Of Activated T Cells 1
*PRDM1*	PR/SET Domain 1
*(BLIMP-1)*	(B lymphocyte-induced maturation protein-1)
*SIRT1*	Sirtuin 1
*TOX*	Thymocyte Selection Associated High Mobility Group Box
*TOX2*	TOX High Mobility Group Box Family Member 2
*TBX21*	T-Box Transcription Factor 21
*(T-bet)*	(T-Box Expressed In T Cells)

**Table 3:** Innate and acquired immune cell markers.

Gene Symbol	Protein name
*CCR7*	C-C Motif Chemokine Receptor 7
*CD3D*	CD3d Molecule
*CD3E*	CD3e Molecule
*CD3G*	CD3g Molecule
*CD4*	CD4 Molecule
*CD8A*	CD8a Molecule
*CD19*	CD19 Molecule
*CD79A*	CD79a Molecule
*CR2*	Complement C3d Receptor 2
*(CD21)*	(CD21 Antigen)
*FAS*	Fas Cell Surface Death Receptor
*(CD95)*	(CD95 Antigen)
*GATA3*	GATA Binding Protein 3
*IL7R*	Interleukin 7 Receptor
*(CD127)*	(CD127 Antigen)
*KLRD1*	Killer Cell Lectin Like Receptor D1
*(CD94)*	(CD94 Antigen)
*LAIR1*	Leukocyte Associated Immunoglobulin Like Receptor 1
*MS4A1*	Membrane Spanning 4-Domains A1
*(CD20)*	(CD20 Antigen)
*NCR1*	Natural Cytotoxicity Triggering Receptor 1
*(NKp46)*	(Natural Killer Cell P46-Related Protein)
*NCR2*	Natural Cytotoxicity Triggering Receptor 2
*(NKp44)*	(Natural Killer Cell P44-Related Protein)
*NCR3*	Natural Cytotoxicity Triggering Receptor 3
*(NKp30)*	(Natural Killer Cell P30-Related Protein)
*PRF1*	Perforin 1
*RORC*	RAR Related Orphan Receptor C
*(RORγt)*	(Nuclear Receptor ROR-Gamma)
*SDC1*	Syndecan 1
*(CD138)*	(CD138 Antigen)

For each CRC sample, the Z-score of the expression level for each gene of interest was calculated and compared to the mean of all the samples in the study. Samples with a Z-score ≥ 2 were considered high-expressing, whereas samples with a Z-score < 2 were considered low-expressing. Survival curves were constructed (with CRC cases for which both gene expression and follow-up data were available) using the Kaplan-Meier method and survival differences were analyzed by the Log-rank test, whereas the association between gene expression and CRC molecular subtypes was assessed using Cramér’s V test. Gene co-occurrence analysis was performed by odds ratio (OR) calculation, while Spearman’s correlation coefficient (*ρ*) was used to exclude correlations between gene expression and patients’ age, sex and TNM staging.

To estimate the expression of immune exhaustion genes in the immune cell subsets infiltrating CRC samples, Transcripts Per Million (TPM)-normalized RNA-seq data of the “*Colorectal Adenocarcinoma TCGA PanCancer collection*” were downloaded from http://firebrowse.org and analyzed using CIBERSORTx (13), a computational framework, which accurately infers cell type abundance from the RNA profiles of tissue samples, using specific gene signatures. LM22 was used as signature matrix, that contains 547 genes, which distinguish 22 human hematopoietic cell phenotypes (14), and batch correction option was enabled, to remove technical differences between the signature matrix and RNA-seq data. For each immune exhaustion gene, the Z-score of the expression level in each immune cell subset was calculated and genes with a Z-score ≥ 2 were considered highly expressed.

All statistical tests were evaluated at an *α* level of 0.05, using Stata, version 13 (StataCorp, College Station, TX, USA; RRID : SCR_012763).

### Patients and Samples

To validate bioinformatic data at the protein level, we collected colon tissue samples and clinicopathological profiles of patients (15) who underwent colectomy for CRC, between 2009 and 2013, at the S.S. Annunziata Hospital (Chieti, Italy). Patients had not received immunosuppressive treatments and were free from chronic inflammatory or immune system diseases. Fifty-three patients were selected by matching for sex, age, clinical stage and molecular subtype with CRC patients from the PanCancer collection (**Table 1**). Follow-up time was of 84 months. Subsequently, patients were divided into short-term survivors (23 patients with an overall survival, OS, ≤ 40 months), and long-term survivors (30 patients with an OS > 40 months). This cut-off value was determined from the estimated survival probabilities derived from the Kaplan-Meier curves of patients from the PanCancer collection.

The study was reviewed and approved by the Ethical Committee of the “G. d’Annunzio” University and Local Health Authority of Chieti, Italy. The study was performed, after written informed consent from patients, in accordance with the principles outlined in the Declaration of Helsinki.

### Histopathology, Immunohistochemistry and Morphometric Analyses

CRC samples, obtained from the validation cohort, based on standard sampling protocols (16), were fixed in 4% formalin and embedded in paraffin. For histology, paraffin-embedded samples were sectioned at 4 μm and stained with hematoxylin and eosin (H&E). Single or double (T-bet/EOMES, PD-1/LAG3, CD20/FCRL4, CD20/BTLA, CD20/TRAF1, CD20/SIGLEC6, CD3/PD-1, CD3/EOMES, CD3/T-bet, CD3/PD-L1, CD3/LAG3, CD3/TGFβ1, NKp46/TIGIT, NKp46/IDO1, NKp46/PD-L2) immunostainings, on formalin fixed and paraffin-embedded tissue sections, were performed as described (17), using the Abs listed in **Table 4**.

**Table 4:** Antibodies used in immunostaining.

Antibody	Clone	Origin	Code	Source
BTLA	EPR22224-271	Rabbit	ab230976	Abcam, Cambridge, UK
CD3		Rabbit	A0452	Agilent, Santa Clara, CA, USA
CD20	L26	Mouse	M0755	“
EOMES	BLR104H	Rabbit	ab275960	Abcam, Cambridge, UK
FCRL4	EPR21961	Rabbit	ab239076	“
IDO1	GT273	Mouse	GTX634652	Genetex, Irvine, CA, USA
LAG3	EPR4392	Rabbit	ab180187	Abcam, Cambridge, UK
NKp46	195314	Mouse	MAB1850	R&D Systems, Minneapolis, MN, USA
PD-1	NAT105	Mouse	ab52587	Abcam, Cambridge, UK
PD-L1	E1L3N	Rabbit	13684	Cell Signaling, Danvers, MA, USA
PD-L2		Rabbit	SAB3500395	Merck, Darmstadt, D
SIGLEC6		Rabbit	ab224406	Abcam, Cambridge, UK
T-bet	O4-46	Mouse	561263	BD Biosciences, Franklin Lakes, NJ, USA
TGFβ1		Rabbit	Sc-146	Santa Cruz, Dallas, TX, USA
TIGIT	BLR047F	Rabbit	A700-047	Bethyl Labs, Montgomery, TX, USA
TRAF1	TRAF1/2770	Mouse	ab268244	Abcam, Cambridge, UK

The morphometric analysis of immune exhaustion markers was confined to the neoplastic areas of colon tissue sections, and was performed by light microscopy, at ×400, in an 85431.59 μm^2^ field, on single or double immunostained sections, with Qwin image analysis software (version 2.7) (Qiagen, Hilden D). The Qwin image analyses ensures the following highly reproducible steps: 1) image acquisition; 2) conversion of RGB image (true colors) to binary image (black and white); 3) filtering to remove noise; 4) counting of immunostained cells or measurement of positively stained area. Six to eight high-power fields, randomly chosen, were evaluated for each section and two sections per sample were analyzed. Results were expressed as mean percentage ± standard deviation (SD) of positive cells/number of total cells per field. Immunostained sections were examined by two pathologists in a blind fashion, with very good agreement (κ value = 0.89). Representative images of immunostained sections for exhaustion markers were captured in the stroma, between tumor glands, or in lymphoid aggregates, because in these areas their expression was more evident, regardless of the number of lymphoid-like structures in tumor samples from short *versus* long-term survivors, which has been the subject of previous investigations (18, 19).

### Statistics

For the bioinformatics, statistical analyses have been described above. For morphometric studies, between-group differences were assessed by Student’s *t*-test. All statistical tests were evaluated at an α level of 0.05, using Stata, version 13 (Stata Corp).

## Results

### Association of the Expression of Immune Exhaustion Genes With the Molecular CRC Subtypes and With the Clinical Outcome

Functional and exhausted immune cells display distinct transcriptional programs (7, 20). To investigate immune cell dysfunction in the CRC microenvironment, we performed bioinformatic analysis of the immune exhaustion gene expression according to the clinic-pathological profiles of CRC patients. For this purpose, we used the publicly available RNA-seq data of CRC samples, obtained from 594 patients (**Table 1**), from the TCGA PanCancer collection (21). The range of genes, whose expression data was analyzed, includes *inhibitory receptors*, *immunosuppressive mediators*, and *transcription factors*, as listed in the **Table 2**. Biostatistical findings, based on RNA-seq data, were then assessed, at the protein expression level, by immunohistochemical and morphometric analyses of CRC samples obtained from a validation cohort of 53 patients.

#### a. High Levels of *PD-L1*, *LAG3* and *T-bet* Expression Are Associated With the MSI Subtype in CRC

Approximately 15% of all CRCs have high frequency of MSI, which results from impaired DNA mismatch repair due to mutation or hypermethylation of mismatch repair genes (*MLH1, MSH2, MSH6* or *PMS2*) (22). This defect leads to the accumulation of insertions and deletions in DNA repeat sequences (microsatellites), that ultimately result in high tumor mutational burden (TMB), and to a high density of CD8^+^ tumor infiltrating lymphocytes, that are required for response to immunotherapy (23). Bioinformatic analyses of gene expression data from CRC samples of TCGA PanCancer collection, revealed that among immune exhaustion-related genes, the expression of those coding for *PD-L1, LAG3* and *T-bet* was associated (Cramér’s V=0.3) with MSI/dMMR tumors, which were diagnosed in 63/594 (10.61%) of CRC patients (**Table 5**).

**Table 5:** Association of the expression of immune exhaustion genes with the molecular subtypes of CRC.

Immune Exhaustion Genes			Molecular Subtypes of CRC*				
			CIN	GS	MSI	POLE	Total
**PD-L1^†^**		**High**	1	0	8	2	11
		**Low**	327	58	55	8	448
			328	58	63	10	459
**LAG3^‡^**		**High**	1	1	11	1	14
		**Low**	327	57	52	9	445
			328	58	63	10	459
**T-bet^§^**		**High**	3	1	10	1	15
		**Low**	325	57	53	9	444
			328	58	63	10	459

*CIN, Chromosomal instability; GS, Genomically stable; MSI, Microsatellite instability; POLE, Polymerase epsilon gene mutation.

^†^Cramer’s V = 0.328; ^‡^Cramer’s V = 0.3448; ^§^Cramer’s V = 0.3.

#### b. High Levels of PD-1, LAG3, T-bet, EOMES, TRAF1 and PD-L1 Expression Are Associated With Shorter Overall Survival in CRC Patients

**Figure 1:**
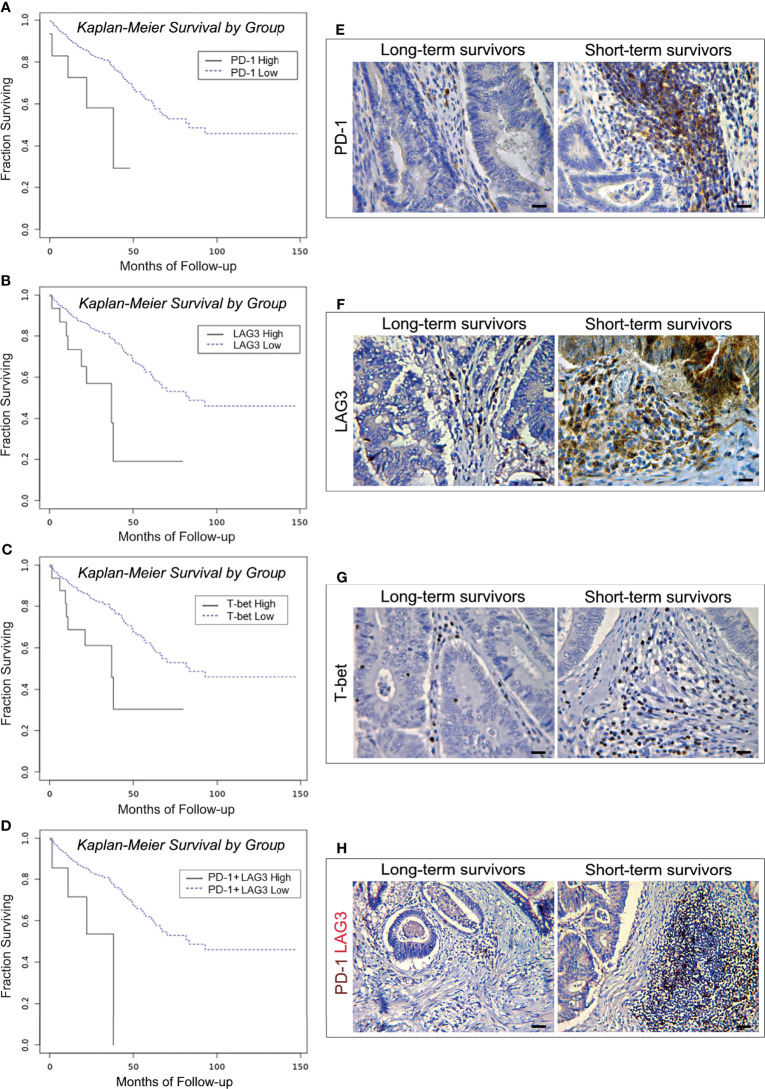
Survival curves and immunopathological features of PD-1, LAG3 and T-bet expression in CRC patients from the PanCancer collection and from the validation cohort. **(A)** Kaplan-Meier curves representing, for each time point, the fraction of surviving CRC patients classified, based on mRNA expression levels in tumor samples, as PD-1 mRNA^high^ (n. 15) and PD-1 mRNA^low^ (n. 573). **(B)** Kaplan-Meier curves representing, for each time point, the fraction of surviving CRC patients classified, based on mRNA expression levels in tumor samples, as LAG3 mRNA^high^ (n. 19) and LAG3 mRNA^low^ (n. 569). **(C)** Kaplan-Meier curves representing, for each time point, the fraction of surviving CRC patients classified, based on mRNA expression levels in tumor samples, as T-bet mRNA^high^ (n. 18) and T-bet mRNA^low^ (n. 570). **(D)** Kaplan-Meier curves representing, for each time point, the fraction of surviving CRC patients classified, based on mRNA expression levels in tumor samples, as PD-1 mRNA^high^ + LAG3 mRNA^high^ (n. 10) and PD-1 mRNA^low^ + LAG3 mRNA^low^ (n. 578). **(E)** Expression of PD-1 in CRC tissue sections obtained from patients with long- and short-term survival. Magnification: X400. Scale bars: 20 μm. **(F)** Expression of LAG3 in CRC tissue sections obtained from patients with long- and short-term survival. Magnification: X400. Scale bars: 20 μm. **(G)** Expression of T-bet in CRC tissue sections obtained from patients with long- and short-term survival. Magnification: X400. Scale bars: 20 μm. **(H)** Co-expression of PD-1 (brown) and LAG3 (red) in CRC tissue sections obtained from patients with long- and short-term survival. Magnification: X200. Scale bars: 30 μm.

To assess whether the expression of *PD-1, LAG3* and *T-bet* in CRC samples, was related with patients’ clinical outcome, Kaplan Meier survival analysis was performed for patients with CRC, of all stages and molecular subtype. As shown in **Figures 1A–C**, the log-rank test indicated that high levels of expression of all these immunity genes were associated with a shorter mean overall survival (OS). Patients bearing tumors with high level of expression of *PD-1* (PD-1^high^) had an OS of 29.43 months, *versus* 91.21 months of patients bearing tumors with a low level of expression (PD-1^low^) (log-rank test, *p*=0.0069).

The OS of patients bearing LAG3^high^, or T-bet^high^ was 34.68 and 39.82 months, respectively, *versus* 91.63 and 91.45 months of patients with LAG3^low^, or T-bet^low^ tumors (log-rank test, *p*=0.0014 and *p*=0.0043, respectively). Odds ratio calculation revealed that the expression of *LAG3* was strongly associated with that of *PD-1* (OR >3, *p*<0.001). In agreement with this finding, high levels of co-expression of these genes were also associated with a shorter OS (26.08 *versus* 91.21 months. Log-rank test, *p*=0.0041) (**Figure 1D**).

To assess whether the association between the expression of immune exhaustion genes in CRC and clinical outcome could be confirmed, at the protein expression level, immunopathological analyses were performed on tumor samples obtained from a validation cohort of 53 CRC patients (matched for sex, age, clinical stage and molecular subtype, with CRC patients from the PanCancer collection, previously analyzed for gene expression data and clinical outcome), which were distinguished, by outcome, as short-term (OS ≤ 40 months, 23 patients) and long-term (OS > 40 months, 30 patients) survivors (**Table 6**).

**Table 6:** Expression of immune exhaustion markers in the validation cohort.

	Short OS*	Long OS*	Student’s *t*-test
	(≤40 months)	(>40 months)	(*p*-value)
**BTLA**	17.04 ± 2.27	2.27 ± 1.36	<0.0001
**EOMES**	24.61 ± 2.98	8.10 ± 1.79	<0.0001
**FCRL4**	14.26 ± 2.56	5.10 ± 2.06	<0.0001
**LAG3**	19.04 ± 4.03	6.60 ± 1.96	<0.0001
**PD-1**	15.91 ± 4.68	5.10 ± 1.83	<0.0001
**PD-L1**	22.30 ± 3.96	4.40 ± 1.71	<0.0001
**SIGLEC6**	9.57 ± 1.83	2.07 ± 1.01	<0.0001
**TRAF1**	23.00 ± 3.22	6.13 ± 1.57	<0.0001
**T-bet**	12.70 ± 1.92	4.60 ± 1.94	<0.0001
**CD20/FCRL4**	11.13 ± 1.82	3.63 ± 1.40	<0.0001
**EOMES/T-bet**	9.26 ± 1.66	3.27 ± 1.91	<0.0001
**PD-1/LAG3**	12.22 ± 1.83	4.67 ± 1.75	<0.0001

OS, overall survival.

*Values are expressed as mean percentages ± standard deviation (SD) of positive cells/number of total cells, evaluated on six-eight high-power fields for each section (two sections per sample were analyzed).

Expression of *PD-1, LAG3* and *T-bet* was detected in lymphoid aggregates and scattered among the neoplastic glands, or at intra-epithelial sites, whereas *LAG3* was also occasionally expressed by clusters of colonic cancer cells (**Figures 1E–G**). Morphometric evaluation revealed that, the mean percentage of immune cells positive for PD-1, or LAG3 or T-bet was higher (*p*<0.001) in CRC samples from short-term survivors, than in tumor samples from long-term survivors, as reported in the **Table 6**.

Double immunostaining of PD-1/LAG3 confirmed their frequent co-expression and showed that tumors obtained from short-term survivors contained a higher percentage (*p*<0.0001) of double positive cells than tumors from long-term survivors (**Figure 1H**, and **Table 6**).

**Figure 2:**
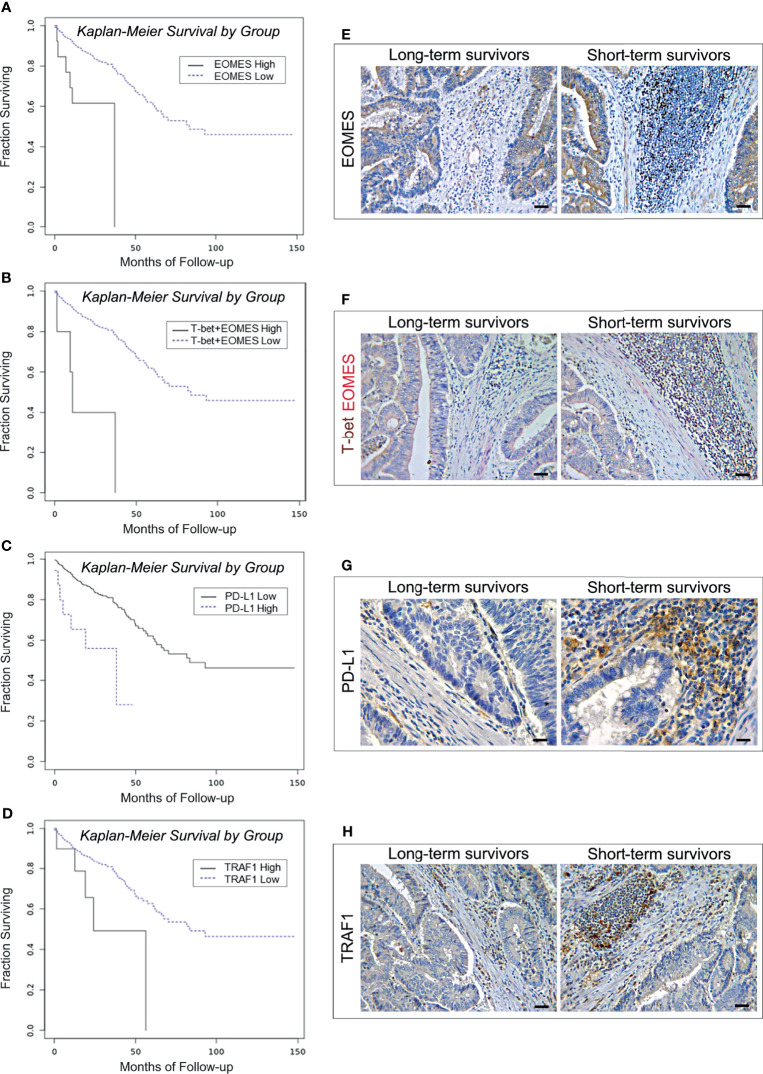
Survival curves and immunopathological features of EOMES/T-bet, PD-L1 and TRAF1 expression in CRC patients from the PanCancer collection and from the validation cohort. **(A)** Kaplan-Meier curves representing, for each time point, the fraction of surviving CRC patients classified, based on mRNA expression levels in tumor samples, as EOMES mRNA^high^ (n. 14) and EOMES mRNA^low^ (n. 574). **(B)** Kaplan-Meier curves representing, for each time point, the fraction of surviving CRC patients classified, based on mRNA expression levels in tumor samples, as T-bet mRNA^high^ + EOMES mRNA^high^ (n. 6) and T-bet mRNA^low^ + EOMES mRNA^low^ (n. 582). **(C)** Kaplan-Meier curves representing, for each time point, the fraction of surviving CRC patients classified, based on mRNA expression levels in tumor samples, as PD-L1 mRNA^high^ (n. 18) and PD-L1 mRNA^low^ (n. 570). **(D)** Kaplan-Meier curves representing, for each time point, the fraction of surviving CRC patients classified, based on mRNA expression levels in tumor samples, as TRAF1 mRNA^high^ (n. 11) and TRAF1 mRNA^low^ (n. 577). **(E)** Expression of EOMES in CRC tissue sections obtained from patients with long- and short-term survival. Magnification: X200. Scale bars: 30 μm. **(F)** Co-expression of T-bet (brown) and EOMES (red) in CRC tissue sections obtained from patients with long- and short-term survival. Magnification: X200. Scale bars: 30 μm. **(G)** Expression of PD-L1 in CRC tissue sections obtained from patients with long- and short-term survival. Magnification: X400. Scale bars: 20 μm. **(H)** Expression of TRAF1 in CRC tissue sections obtained from patients with long- and short-term survival. Magnification: X200. Scale bars: 30 μm.

Expanding the analysis to the whole range of immune exhaustion markers (**Table 2**) has revealed that high levels of expression of *TRAF1* and *PD-L1* were also associated with worst patient outcome. The OS of patients bearing EOMES^high^ tumors was 25.16 months, *versus* 91.34 months for patients bearing EOMES^low^ tumors (log-rank test, *p*=0.0003; **Figure 2A**). OR calculation revealed a strong association between the expression of *EOMES* and expression of *T-bet* (OR >3, *p*<0.001). Notably, co-expression of high levels of *T-bet* and *EOMES* led to a further reduction in the OS (19.17 *versus* 91.18 months for patients with low co-expression levels (log-rank test, *p*<0.0001; **Figure 2B**). Finally, patients bearing PD-L1^high^ or TRAF1^high^ CRCs, had a shorter OS (27.34 months and 35.70 months, respectively), than patients bearing PD-L1^low^ or TRAF1^low^ CRCs (91.44 and 91.60 months, respectively. Log-rank test, *p*=0.0004 and *p*=0.0188, respectively; **Figures 2C, D**).

Immunohistochemistry corroborated the biostatistical findings showing that CRC samples, from short-term survivors, contained an average percentage of EOMES/T-bet double positive cells, and of PD-L1 and TRAF1 positive cells, that was considerably higher (*p*<0.0001) than in CRC samples from long-term survivors (**Table 6** and **Figures 2E–H**).

#### c. High Levels of *BTLA, FCRL4* and *SIGLEC6* Are Associated With Shorter Overall Survival in CRC Patients

B cell exhaustion was first described in association to HIV infection, after the demonstration of persistent virus-induced T-cell exhaustion (24). Inhibition of B cell receptor (BCR) signaling, and loss of B cell effector functions, are tightly regulated by several different co-receptors (9, 24). CD22 (SIGLEC2), PD-1, BTLA, LAIR1, FCRL4 and CD327 (SIGLEC6) have been identified as inhibitors of BCR signaling (9, 25, 26), and therefore their expression was analyzed to assess the functional state of B cells in CRC samples of the PanCancer collection. Biostatistics revealed that the transcriptional expression of genes coding for *BTLA* (27), *FCRL4* (28) and for *SIGLEC6* (29), was associated with worst patient outcome.

**Figure 3:**
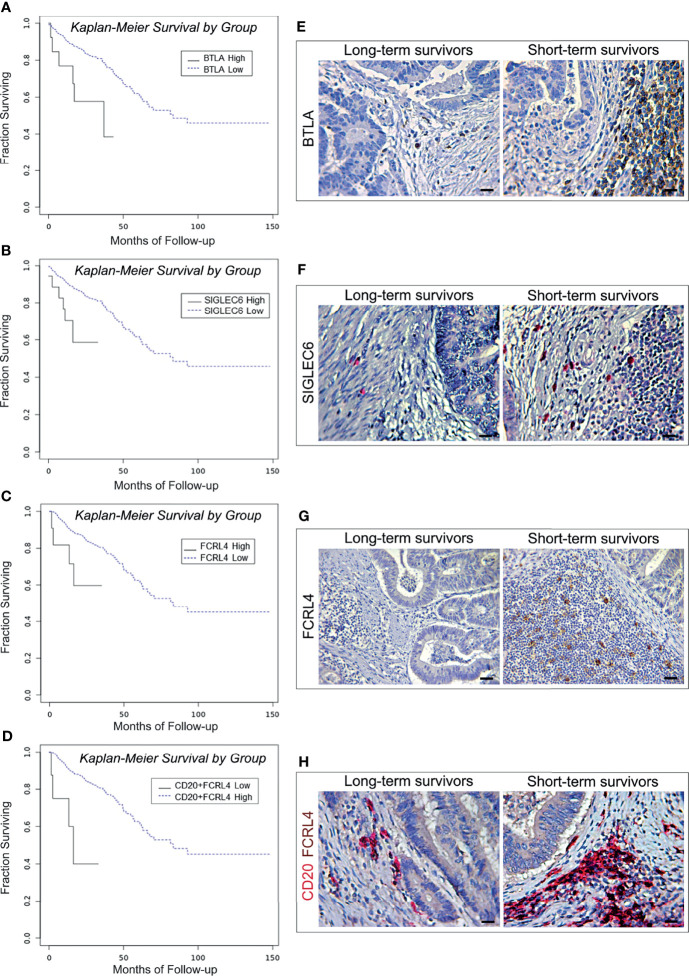
Survival curves and immunopathological features of BTLA, SIGLEC6, and CD20/FCRL4 expression in CRC patients from the PanCancer collection and from the validation cohort. **(A)** Kaplan-Meier curves representing, for each time point, the fraction of surviving CRC patients classified, based on mRNA expression levels in tumor samples, as BTLA mRNA^high^ (n. 15) and BTLA mRNA^low^ (n. 573). **(B)** Kaplan-Meier curves representing, for each time point, the fraction of surviving CRC patients classified, based on mRNA expression levels in tumor samples, as FCRL4 mRNA^high^ (n. 11) and FCRL4 mRNA^low^ (n. 351). **(C)** Kaplan-Meier curves representing, for each time point, the fraction of surviving CRC patients classified, based on mRNA expression levels in tumor samples, as SIGLEC6 mRNA^high^ (n. 18) and SIGLEC6 mRNA^low^ (n. 570). **(D)** Kaplan-Meier curves representing, for each time point, the fraction of surviving CRC patients classified, based on mRNA expression levels in tumor samples, as CD20 mRNA^high^ + FCRL4 mRNA^high^ (n. 8) andCD20 mRNA^low^ + FCRL4 mRNA^low^ (n. 354). **(E)** Expression of BTLA in CRC tissue sections obtained from patients with long- and short-term survival. Magnification: X400. Scale bars: 20 μm. **(F)** Expression of SIGLEC6 in CRC tissue sections obtained from patients with long- and short-term survival. Magnification: X400. Scale bars: 20 μm. **(G)** Expression of FCRL4 in CRC tissue sections obtained from patients with long- and short-term survival. Magnification: X200. Scale bars: 30 μm. **(H)** Co-expression of CD20 (red) and FCRL4 (brown) in CRC tissue sections obtained from patients with long- and short-term survival. Magnification: X400. Scale bars: 20 μm.

Specifically, the OS of patients bearing BTLA^high^ tumors was 27.82 months, *versus* 91.27 months for patients bearing BTLA^low^ tumors (log-rank test, *p*=0.0052; **Figure 3A**). The OS of patients bearing CRC with high level of expression of *SIGLEC6* or *FCRL4* was 23.08 and 24.54 months, respectively, *versus* 91.04 and 91.30 months for patients bearing tumors with low expression levels (log-rank test, *p*=0.0033 and *p*=0.0303, respectively; **Figures 3B, C**). Notably, co-expression of high levels of *FCRL4* and *CD20* in CRC samples was associated with a shorter OS, compared to low transcriptional levels of both genes (18.91 *versus* 91.48 months. Log-rank test, *p*=0.0007 and **Figure 3D**).

Morphometric analyses performed on CRC samples from the validation cohort substantiated the survival data of patients from the PanCancer collection, revealing that the mean percentage of BTLA and SIGLEC6 positive cells was significantly (*p<*0.0001) higher in short-term survivors than in long-term survivors. Moreover, immunostaining for FCRL4 and double staining for both CD20 and FCRL4 performed on CRC samples from short-term survivors, also showed mean percentages of single or double positive cells significantly (*p*<0.0001) higher than that found in CRC samples from long-term survivors (**Table 6** and **Figures 3E–H**).

### Association of Immune Exhaustion Genes With T and B Lymphocyte Markers, and Conventional NK Cell Markers in CRC Samples

**Figure 4:**
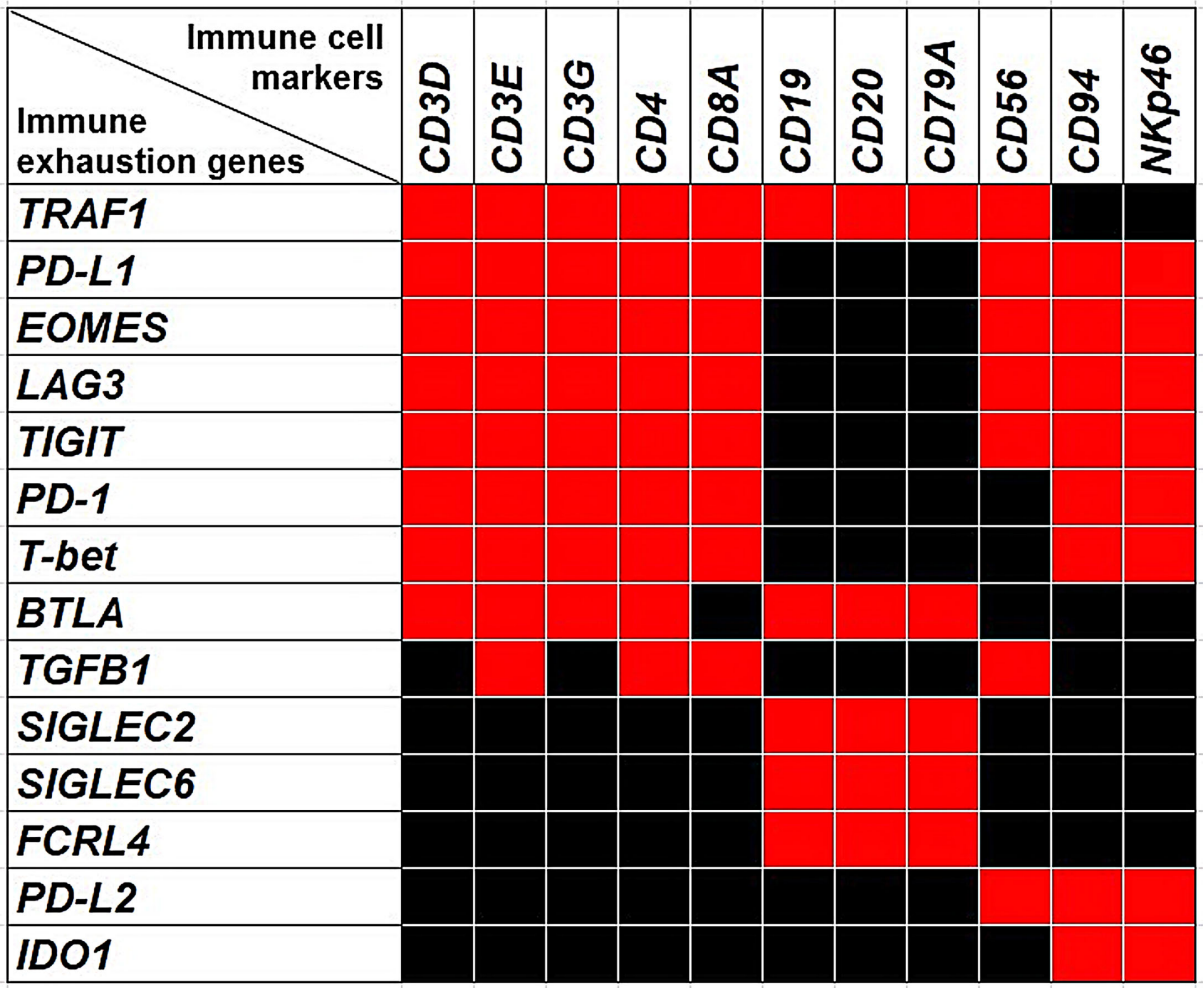
The Heatmap shows the associations between the expression of immune exhaustion genes and the expression of immune cell markers. Red squares indicate co-occurrence (OR >1.5, *p*<0.05), while black squares indicate lack of association.

Since the expression of many of the genes that regulate functional exhaustion can be shared by multiple immune cell types (30), to bring out the immune populations most involved in the functional exhaustion that occurs in CRC, we next investigated, by OR calculation (**Figure 4**), if there was an association between the transcriptional expression of each exhaustion gene and immune cell marker genes, which are listed in the **Table 3**.

#### a. Expressions of *PD-L1, PD-1, LAG3, EOMES, T-bet, TIGIT, TRAF1, BTLA* and *TGFB1* Are Associated With T Lymphocyte Markers

Expressions of *TIGIT, T-bet, EOMES, LAG3, PD-1* and *PD-L1* were significantly (*p*<0.05) correlated with each other and were associated with the expression of genes coding for *CD4, CD8a*, and *CD3γ, CD3δ* and *CD3ε* chains, with an OR >1.5; *p*<0.05, which suggests that expression of these immune exhaustion genes primarily involves *CD8^+^* and *CD4^+^CD3^+^* T cells. Furthermore, expression of *TRAF1* co-occurred with those of *PD-1, PD-L1, EOMES, T-bet, TIGIT, BTLA, CD3γ, CD3δ, CD3ε, CD4* and *CD8a*, with an OR >2, *p*<0.05.

*BTLA* co-occurred with the expression of *PD-1, PD-L1, EOMES, T-bet, TIGIT, TRAF1 CD3γ, CD3δ, CD3ε* and *CD4* (OR >1.5; *p*<0.05), whereas *TGFβ1* co-occurred with *PD-1, PD-L1, LAG3, CD3γ, CD8a*, and *CD4* (OR >2; *p*<0.05).

**Figure 5:**
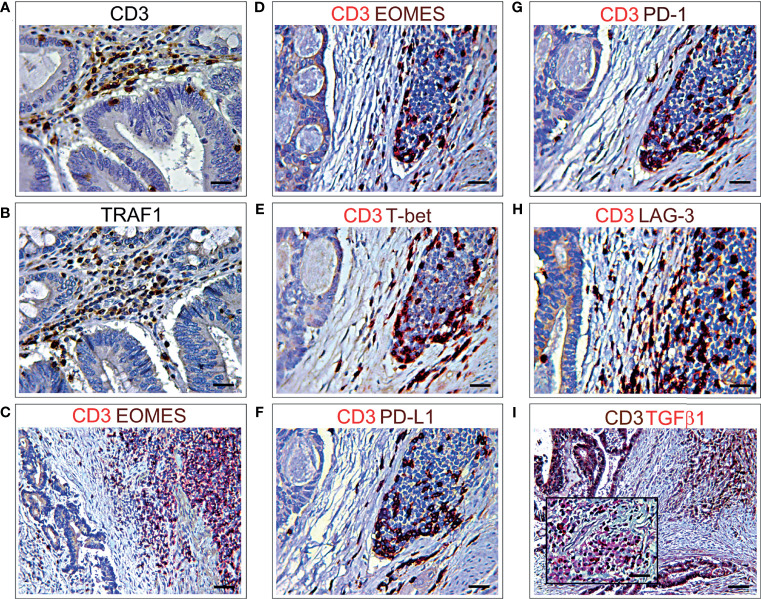
Immunohistochemical features of CD3^+^ T lymphocyte co-localization with different immune exhaustion molecules in CRC. **(A)** Immunohistochemical staining of CRC tissue sections, from short-term survivors, showing CD3^+^ T cells in the stroma, among neoplastic glands. Magnification: X400. Scale bars: 20 μm. **(B)** Immunohistochemical staining of consecutive serial CRC tissue sections, from short-term survivors, showing TRAF1^+^ cells localized in the areas infiltrated by CD3^+^ T cells, among neoplastic glands. Magnification: X400. Scale bars: 20 μm. **(C, D)**. Double immunohistochemical staining of CRC tissue sections from short-term survivors shows a frequent co-localization of EOMES (brown) with CD3 (red). Images C and D represent two different areas of the same sample. Magnification: C, X200; D, X400. Scale bars: C, 30 μm; D, 20 μm. **(E)** Double immunohistochemical staining of CRC tissue sections from short-term survivors shows a frequent co-localization of T-bet (brown) with CD3 (red). Magnification: X400. Scale bars: 20 μm. **(F)** Double immunohistochemical staining of CRC tissue sections from short-term survivors shows that the vast majority of CD3^+^ cells (red) express PD-L1 (brown). Magnification: X400. Scale bars: 20 μm. **(G)** Double immunohistochemical staining of CRC tissue sections from short-term survivors shows that the vast majority of CD3^+^ cells (red) express PD-1 (brown). Magnification: X400. Scale bars: 20 μm. **(H)** Double immunohistochemical staining of CRC tissue sections from short-term survivors shows that most of CD3^+^ cells (red) express LAG3 (brown). Magnification: X400. Scale bars: 20 μm. **(I)** Double immunohistochemical staining of CRC tissue sections from short-term survivors shows a frequent co-localization of TGFβ1 (red) with CD3^+^ cells (brown). Magnification of CD3^+^TGFβ1^+^ inflammatory infiltrate in the inset. Magnification: X200; Inset, X400. Scale bars: 30 μm; Inset, 20 μm.

To evaluate, at the level of protein expression, the gene expression data provided by bionformatics, CD3 and TRAF1 immunostaining of consecutive serial sections, and double immunostainings of CD3 with PD-L1, or PD-1, or LAG3, or EOMES, or T-bet, or TIGIT or TGFβ1 were performed on CRC samples from the short-term survivors, which revealed a higher level of immune exhaustion. The frequent co-localizations of these immune exhaustion molecules with CD3^+^ cells, in the lymphoid aggregates, or scattered in the stroma and CRC epithelia, substantiated the biostatistical findings (**Figures 5A–I**).

Furthermore, to assess the immune exhaustion-related gene expression by the different T lymphocyte subpopulations, we used CIBERSORTx, which, when applied to the PanCancer collection data, was able to detect, in the context of CRC microenvironment, the expression of FOXP1 and SIRT1 in CD4^+^ memory resting and CD8^+^ cells. CD8^+^ cells also expressed BATF, whereas CD4^+^ memory resting cells also expressed NR4A1 and TOX.

Interestingly, CIBERSORTx analyses revealed that expression of FOXP1 was not confined to the T cell populations, including T regulatory (Tregs) cells, but also involved both naïve and memory B cells, and that expression of SIRT1 and NR4A1 was extended to naïve B cells, whereas expression of BATF was also found in resting NK cells.

#### b. Expressions of *BTLA, FCRL4, SIGLEC2, SIGLEC6* and *TRAF1* Are Associated With B Lymphocyte Markers

**Figure 6:**
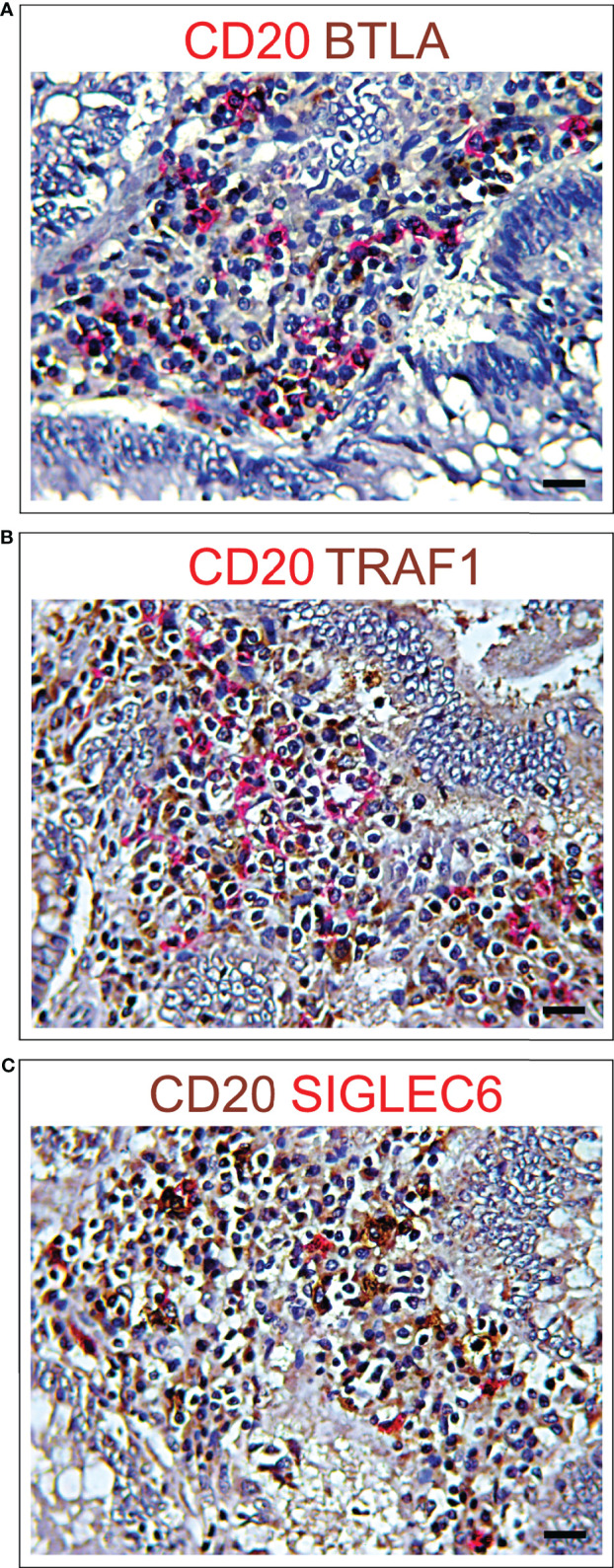
Immunohistochemical features of CD20^+^ B lymphocyte co-localization with different immune exhaustion molecules in CRC. **(A)** Double immunohistochemical staining of CRC tissue sections from short-term survivors shows a frequent co-localization of BTLA (brown) with CD20 (red). Magnification: X400. Scale bars: 20 μm. **(B)** Double immunohistochemical staining of CRC tissue sections from short-term survivors shows a frequent co-localization of TRAF1 (brown) with CD20 (red). Magnification: X400. Scale bars: 20 μm. **(C)** Double immunohistochemical staining of CRC tissue sections from short-term survivors shows a frequent co-localization of SIGLEC6 (red) with CD20 (brown). Magnification: X400. Scale bars: 20 μm.

Expressions of *BTLA, FCRL4* and *SIGLEC6*, that were found to be associated with a worst outcome (**Figures 3A–D**), correlated with each other and with the expression of *SIGLEC2* (OR >2.5, *p*<0.05), which also dampens BCR signaling (26). It is noteworthy that, the expression of *BTLA, FCRL4, SIGLEC2* and *SIGLEC6* genes, showed a moderate co-occurrence with the expression of typical B cell markers, such as *CD19, CD20* and *CD79a* with an OR >2.5, *p*<0.05, and immunohistochemistry clearly showed the frequent co-localization of BTLA, FCRL4 and SIGLEC6 with CD20^+^ cells (**Figures 3H**, **6A, C**). As observed for *BTLA*, the expression of *TRAF1* not only co-occurred with T cell markers, but also with the expression of *CD19, CD20* and *CD79a* (OR >2, *p*<0.05), which was confirmed by immunohistochemistry (**Figures 5A, B**, **6B**), thus suggesting its contribution to B cell exhaustion.

#### c. Expression of *PD-L1, PD-L2, PD-1, LAG3, TIGIT* and *IDO1* Is Associated With Conventional NK Cell Markers

Further insights into the immune exhaustion profiles of CRCs from the PanCancer collection, revealed that expression of genes coding for *PD-L1, PD-1, LAG3*, and *TIGIT* not only co-occurred with those coding for T cell markers, but along with the expression of *PD-L2* (31), and *IDO1* (32, 33), also co-occurred with the expression of genes that identify a subset of group 1 innate lymphoid cells (ILC) defined as conventional (c) NK cells (34), which are endowed with high level expression of transcription factors, *EOMES* and *T-bet*, and surface molecules, *NKp46* and *CD94/NKG2A*. The OR for the co-occurrence of *PD-L1, PD-1, LAG3, TIGIT, PD-L2, EOMES, T-bet, NKp46* and *CD94/NKG2A* was > 1.5; *p*<0.05.

**Figure 7:**
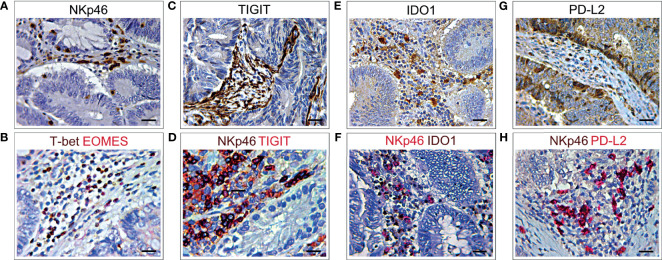
Immunohistochemical features NKp46 co-localization with different immune exhaustion molecules in CRC. **(A)** Immunohistochemical staining of CRC tissue sections, from short-term survivors, showing NKp46^+^ cells in the stroma, among neoplastic glands. Magnification: X400. Scale bars: 20 μm. **(B)** Double immunohistochemistry of consecutive serial CRC tissue sections, showing that EOMES (red) frequently co-localized with T-bet (brown). EOMES^+^T-bet^+^ cells (brick red) localized in the stroma, among neoplastic glands, in the same area showing NKp46^+^ cell infiltrate **(A)**. Magnification: X400. Scale bars: 20 μm. **(C)** Immunohistochemical staining of consecutive serial CRC tissue sections, showing TIGIT^+^ cells localized in the stroma, among neoplastic glands. Magnification: X400. Scale bars: 20 μm. **(D)** Double immunohistochemistry of consecutive serial CRC tissue sections, showing that TIGIT (red) frequently co-localizes with NKp46 cells (brown), as revealed by the brick red stained NKp46^+^TIGIT^+^ cells. Magnification: X400. Scale bars: 20 μm. **(E)** Immunohistochemical staining of consecutive serial CRC tissue sections showing expression of IDO1 in the stroma, among neoplastic glands. Magnification: X400. Scale bars: 20 μm. **(F)** Double immunohistochemistry of consecutive serial CRC tissue sections, showing that IDO1 (brown) frequently co-localizes with NKp46 cells (red), as revealed by the brick red stained NKp46^+^IDO1^+^ cells. Magnification: X400. Scale bars: 20 μm. **(G)** Immunohistochemical staining of consecutive serial CRC tissue sections showing PD-L2 expression in stromal infiltrating cells and colon cancer cells. Magnification: X400. Scale bars: 20 μm. **(H)** Double immunohistochemistry of consecutive serial CRC tissue sections, showing that PD-L2 (red) frequently co-localizes with NKp46 cells (brown), as revealed by the brick red stained NKp46^+^PD-L2^+^ cells. Magnification: X400. Scale bars: 20 μm.

Immunostainings of consecutive (3 μm serial) CRC tissue sections, from short-term survivors of the validation cohort, revealed a distinct expression of TIGIT, IDO1 and PD-L2 among the neoplastic glands, mostly in the areas infiltrated by EOMES^+^T-bet^+^NKp46^+^ cells. However, expression of TIGIT extended to other stromal infiltrating cells, whereas PD-L2 was also expressed by colorectal cancer cells (**Figures 7A–H**).

Taken together, immunohistochemical and morphometric analyses substantiated the biostatistical findings, which suggested that CRC-associated immune exhaustion, demonstrated by high levels of a wide range of immune checkpoint molecules, affects T and B lymphocytes, and cNK cells, and is associated with a worse clinical outcome, independently of the disease stage and molecular subtype.

## Discussion

Despite the survival benefit of early detection through increased screening, CRC remains a leading cause of cancer related death (35), which is primarily due to disease progression or to the failure of current therapies to counteract metastasis (36). Immunotherapy, which has recently revolutionized the treatment of advanced tumors (37, 38), needs to be improved for CRC care, since: a) eligible patients are limited to a subset diagnosed with mismatch-repair-deficient mutations or microsatellite instability tumors (39) and, b) responsive patients will eventually experience resistance to treatment and relapse (40), concurrently with a weakening of their immune response, namely immune exhaustion (8). Exhaustion, i.e. the progressive loss of effector function due to chronic low-affinity antigen stimulation, is essential in maintaining immune homeostasis by regulating the duration and the magnitude of T cell responses, through a reduced proliferation, impaired effector functions, and elevated and sustained expression of multiple inhibitory receptors, known as immune checkpoint proteins (41). It is becoming progressively clear that the range of checkpoint inhibitors extends well beyond the few targets of current immunotherapy (41), and that functional exhaustion is not an exclusive matter of T lymphocytes, but heavily involves other immune cell populations (12, 42–44), that take part in mounting an effective immune response (19, 45, 46).

Through biostatistics and immunopathological investigations, this study explores the transcriptional program and immune cell context, that characterize the immune exhaustion landscape of CRC. While confirming the previously reported observation of high levels of expression of *PD-L1, LAG3* and *T-bet* in the context of MSI tumors (39, 47, 49), our findings reveal that, independently of tumor stage and molecular subtype, high levels of a wide range of inhibitory receptors and exhaustion-related transcription factors, such as *EOMES, T-bet, PD-L1, PD-1, LAG3, BTLA, FCRL4, SIGLEC6* and *TRAF1* are associated with reduced survival in CRC patients.

TRAF1 belongs to the Tumor necrosis factor receptor (TNFR)-associated factors (TRAF) protein family, which plays important roles in the immune system as key intracellular signaling molecules in TNFR, Toll-like receptor (TLR), cytokine and antigen receptor signaling pathways (19). TRAF1 expression in T and B lymphocytes can be induced by different stimuli that activate the transcription factor NF-κB (49–51), and may act as both positive and negative regulator of immune signaling. Our immunopathological analyses show TRAF1 expression in CRC infiltrating immune cells and lymphoid aggregates, mostly in CRC samples from short-term survivors, coherently with its negative prognostic value. Besides being produced by immune cells, TRAF1 can be overexpressed by neoplastic cells in different B cell malignancies, and in solid tumors including non-small cell lung cancer for which TRAF1 has been proposed as a biomarker of tumor progression and worst clinical outcome (52–54).

Both PD-1, which binds to PD-L1 and PD-L2, and LAG3, which binds to MHC class II molecules, can be found on T and B lymphocytes, macrophages, dendritic cells (DCs), and NK cells (55–59). In CRC their expressions are strongly correlated with each other and, along with TIGIT and BTLA, are tightly associated with markers of T lymphocytes and cNK cells, in which they can inhibit receptor dependent activation (60), thereby dampening anti-tumor effector functions.

BTLA is primarily expressed on B and T cells, and on monocytes and DCs to a lesser extent, and shares structural similarity with PD-1, but its only known ligand is the Herpes virus entry mediator (HVEM), a member of the tumor necrosis factor receptor superfamily (TNFR-SF) (61). The inhibition of T cells by BTLA is stronger than the positive stimulatory effect of HVEM, a co-stimulatory tumor-necrosis factor receptor on T cells, and prevents the excessive activation of T cells (62), thereby maintaining T cell tolerance (63). It is prominently expressed on human T cells in the TME and can inhibit tumor specific CD8^+^ T cells (64). BTLA blockers have been found to enhance human T cell responses when used alone or in combination with antibodies against PD-1 (65–67). Although it is well established that BTLA mainly functions as a negative regulator of lymphocytes, recent studies indicate that the role of BTLA in tumor-resident T cells is complex, as engagement by its ligand, HVEM, inhibits proliferation and cytokine production, but promotes survival of tumor-infiltrating lymphocytes (68). Furthermore, BTLA associates with the BCR and, upon binding to HVEM, recruits the tyrosine phosphatase Src homology 2 domain-containing phosphatase 1, reduces activation of signaling molecules downstream of the BCR and inhibits B cell activation (69).

The role of TGFβ1 in the development of T regulatory cells and immunosuppression is well established (70, 71). In CRC its expression has been found to be increased when compared to adenoma and normal colon tissue (72). Elevated TGFβ1 levels were observed in the primary tumor and in plasma from CRC patients and were correlated with metastasis and poor prognosis (73, 74). TGFβ1 has also been implicated in T cell exhaustion. Notably, inhibition of TGFβ1 can prevent CRC metastasis by unleashing a cytotoxic T cell response against cancer cells, implying that TGFβ1 signaling suppresses cancer recognition by the immune system (75, 76). *In vivo*, inhibition of TGFβ1 signaling in CD8^+^ T cells, through expression of a dominant-negative receptor, improved the function of exhausted T cells (77). Enhanced and sustained TGFβ1/Smad signaling is a distinctive feature of virus-specific CD8^+^ T cells during chronic viral infections *in vivo* and leads to the up-regulation of the pro-apoptotic protein Bim and to T cell apoptosis. By contrast diminished TGFβ receptor signaling reduces T cell death and indirectly inhibits PD-1 and IL10 expression, and enables the acquisition of effector CD8^+^ T cell functions (77).

While PD-L2 expression is mainly restricted to professional APCs, such as DCs and macrophages, PD-L1 is constitutively expressed by T and B cells, macrophages and DCs and is up-regulated by inflammatory mediators (78). However, it can also be expressed by cancer cells in different tumors, including CRC, and inhibit the adaptive immune response preventing tumor cell apoptosis, which could explain the association of its high levels of expression with a worse clinical outcome (79–81).

EOMES and T-bet are T-box transcription factors that drive the differentiation and function of cytotoxic CD8^+^ T lymphocytes and NK cells (82) and that promote type 1 innate and adaptive cell-mediated immunity (83). In HCV and HIV infected patients, EOMES^high^ T cells display high expression of inhibitory receptors and severe functional defects, that lead to the persistent stage of chronic infection (84, 85). In acute myeloid leukemia, it has been reported that EOMES binds to the promoter of TIGIT and up-regulates the expression of this inhibitory receptor on patient-derived T cells. Moreover, in these patients a high frequency of EOMES^+^ T-bet^low^ CD8^+^ T cells was associated with poor response to chemotherapy and shorter OS (86, 87). Co-expression of high levels of EOMES and T-bet, and its correlation with the expression of PD-L1, PD-1, LAG3, and TIGIT has been determined, trough bioinformatic analyses, in CRC from patients with a shorter OS, and confirmed by immunopathological investigations.

TIGIT is a checkpoint receptor which mediates T and NK cell exhaustion in tumor-bearing mice and in patients with colon cancer (88). Immunostainings of CRC samples from short-term survivors show a strong expression of TIGIT in intra-tumoral immune cells, scattered within the cancer and lymphoid structures, but also in fibroblast-like stromal cells, resulting in a robust barrier against immune attack. Blockade of TIGIT, in colon cancer-bearing mice, prevents NK cell exhaustion and promotes a potent tumor-specific T cell immunity in an NK cell-dependent manner (88). Conventional NK cells belong to group 1 ILCs and are essential in killing infected or transformed cells that have null or low expression levels of MHC-I molecules (89, 90). NK cells are involved in the control of CRC progression and metastasis, but they are also susceptible to tumor-directed dysregulation (60, 91, 92). Similar to T cells, NK cells undergo exhaustion during tumor progression or chronic infections and reveal impaired cytolytic activity and production of effector cytokines, downregulation of activating receptors and overexpression of inhibitory receptors (93). Analysis of RNA-seq data from the TCGA PanCancer cohort and morphological investigations of CRC samples from the validation cohort, reveal that expression of PD-L1, PD-1, LAG3, EOMES, T-bet and TIGIT is associated and co-localizes not only with CD8^+^ and CD4^+^CD3^+^ T cells, but as well as with the expression of PD-L2 and IDO1, it co-occurs with typical markers of cNK cells, which are identified as NKp46^+^EOMES^+^T-bet^+^CD94^+^, a subset of group 1 ILCs (34). Group 1 ILCs were found to be increased in CRC, when compared with normal colonic mucosa (94), and to exhibit high level of inhibitory receptor in the late stage of the disease (92). The CD94/NKG2-A functions as a natural killer cell inhibitory receptor for different HLA class I alleles (95) including HLA-E, its predominant ligand, which confers resistance to NK-cell-mediated lysis (96). PD-1 overexpression in NK cells results in decreased degranulation (97), indicating that PD-1 signaling is suppressive not only in T cells but also in NK cells, and identifies a subset of fully mature NK cells with low proliferative response and impaired anti-tumor activity (98). A role as inhibitor of NK effector functions has been recently described also for IDO1, a rate-limiting metabolic enzyme that converts tryptophan into downstream catabolites kynurenines (KYN) (99) and is involved in the establishment and maintenance of peripheral tolerance and tumor immune escape (33). It has been demonstrated that KYN suppresses the function of CD4^+^ T cells and DCs (100) and promotes NK cell apoptosis (101), and that tryptophan-derived L-KYN impairs NK cell cytotoxicity by decreasing NKp46 and NKG2D/NKG2DLs activating receptors (102–104).

A scenario which mimics exhaustion in T lymphocytes, can affect B cells during chronic infection, inflammatory diseases (9, 25, 105) and cancer (106). B cell exhaustion manifests through the progressive loss of effector functions, of antibody and cytokine production, and high levels of inhibitory receptors. BTLA, FCRL4 and SIGLEC6, which have been shown to inhibit BCR signaling (26), have been found to be highly expressed in CRC from patients with shorter OS, by Kaplan Meyer curves of the PanCancer cohort, and in CRC of short-term survivors from the validation cohort, as detected by immunopathology. The tight association of high levels of BTLA, FCRL4, SIGLEC2 and SIGLEC6 (107) with the B lymphocyte markers, CD19, CD20 and CD79a, along with their negative prognostic value, strongly suggests a role for exhausted B lymphocytes in CRC immune escape and patient outcome.

The CIBERSORTx tool, which identifies immune cell subpopulations, based on their molecular signature, provides additional information on the immune exhaustion landscape of CRC, and strengthens the concept that, in the TME, exhaustion affects cells other than just T lymphocytes. Expression of FOXP1 and SIRT1, which can suppress antitumor T cells (108) and promote chemoresistance (109, 110), has been found in CRC infiltrating Tregs (FOXP1), CD8^+^ and memory resting CD4^+^ cells, but also in naïve and memory B lymphocytes (both FOXP1 and SIRT1). Expression of BATF involves CD8^+^ cells (111), but also resting NK cells, while expression of NR4A1 and TOX (112, 113) has been found in memory resting CD4^+^ cells, but also in naïve and memory B cells, respectively.

The integration of bioinformatics with immunopathology, used in the present study, provides new insight into the CRC microenvironment and its clinical relevance. Results suggest that CRC-associated immune exhaustion is not limited to the MSI subtype and is not exclusive of T lymphocytes, but it also involves B cells and cNK cells, and includes a wide range of checkpoint molecules and immune-related transcription factors, with significant prognostic and therapeutic implications. Taken as a whole, our findings emphasize the need to extend genome sequencing and comprehensive immunopathological analyses of individual CRC into the clinical practice, for the development of a multi-targeted patient tailored immunotherapy to effectively counteract disease progression.

## Data Availability Statement

The raw data supporting the conclusions of this article will be made available by the authors, without undue reservation.

## Ethics Statement

The studies involving human participants were reviewed and approved by Ethical Committee of the “G. d’Annunzio” University and Local Health Authority of Chieti. The patients/participants provided their written informed consent to participate in this study.

## Author Contributions

EDC conceived and supervised the study. CS performed bioinformatic analyses. LD’A, CF, and SLC performed the experiments. EDC, CS, and LD’A interpreted the data. EDC wrote the manuscript. All authors contributed to the article and approved the submitted version.

## Funding

The research leading to these results has received funding from AIRC under IG 2019 - ID. 23264 project – P.I. EDC and from the Italian Ministry of University and Research (PRIN - 2017M8YMR8 – Unit 3 P.I. EDC).

## Conflict of Interest

The authors declare that the research was conducted in the absence of any commercial or financial relationships that could be construed as a potential conflict of interest.

## Publisher’s Note

All claims expressed in this article are solely those of the authors and do not necessarily represent those of their affiliated organizations, or those of the publisher, the editors and the reviewers. Any product that may be evaluated in this article, or claim that may be made by its manufacturer, is not guaranteed or endorsed by the publisher.
